# Effects of ****β****_2_ Agonists, Corticosteroids, and Novel Therapies on Rhinovirus-Induced Cytokine Release and Rhinovirus Replication in Primary Airway Fibroblasts

**DOI:** 10.1155/2011/457169

**Published:** 2011-10-24

**Authors:** David Van Ly, Nicholas J. C. King, Lyn M. Moir, Janette K. Burgess, Judith L. Black, Brian G. Oliver

**Affiliations:** ^1^Respiratory Research Group, Discipline of Pharmacology, The University of Sydney, Sydney, NSW 2006, Australia; ^2^Woolcock Institute of Medical Research, Sydney, NSW 2037, Australia; ^3^Discipline of Pathology, The University of Sydney, Sydney, NSW 2006, Australia; ^4^ANZAC Research Institute, Sydney, NSW 2139, Australia

## Abstract

Rhinovirus-(RV-) induced asthma exacerbations account for high asthma-related health costs and morbidity in Australia. The cellular mechanism underlying this pathology is likely the result of RV-induced nuclear-factor-kappa-B-(NF-*κ*B-) dependent inflammation. NF-*κ*B may also be important in RV replication as inhibition of NF-*κ*B inhibits replication of other viruses such as human immunodeficiency virus and cytomegalovirus. To establish the role of NF-*κ*B inhibitors in RV-induced IL- 6 and IL-8 and RV replication, we used pharmacological inhibitors of NF-*κ*B, and steroids and/or *β*_2_ agonists were used for comparison. Primary human lung fibroblasts were infected with RV-16 in the presence of NF-*κ*B inhibitors: BAY-117085 and dimethyl fumarate; *β*_2_ agonist: salmeterol; and/or corticosteroids: dexamethasone; fluticasone. RV-induced IL-6 and IL-8 and RV replication were assessed using ELISAs and virus titration assays. RV replicated and increased IL-6 and IL-8 release. Salmeterol increased, while dexamethasone and fluticasone decreased RV-induced IL-6 and IL-8 (*P<0.05*). The NF-*κ*B inhibitor BAY-117085 inhibited only RV-induced IL-6 (*P<0.05*) and dimethyl fumarate did not alter RV-induced IL-6 and IL-8. Dimethylfumarate increased RV replication whilst other drugs did not alter RV replication. These data suggest that inhibition of NF-*κ*B alone is unlikely to be an effective treatment compared to current asthma therapeutics.

## 1. Introduction

Asthma is a chronic inflammatory disease of the airways characterised by reversible airflow obstruction, inflammation, and hyperresponsiveness to various allergic or non-allergic stimuli such as house dust mites or exercise [1]. 

An asthma exacerbation is the increase in the duration and severity of respiratory symptoms often resulting in hospitalization. Respiratory viruses cause 85% of asthma exacerbations, and 62% of all viral induced asthma exacerbations are caused by human rhinovirus (RV) [2]. Viral-induced asthma exacerbations account for over 50% of the total asthma-related health costs and also increase asthma morbidity. 

The bronchial epithelium has always been considered as the primary site of RV infection; however, increasing *in vivo* evidence shows that RV can also infect submucosal cells such as fibroblasts and airway smooth muscle (ASM) [3, 4]. *In vitro* studies have shown that transformed and primary human airway cells infected with RV release a plethora of proinflammatory cytokines, such as interleukin (IL)-6, IL-8, and the antiviral cytokine: interferon (IFN)-*λ*s [3, 5–7]. The mechanism of this is most likely due to RV-activated nuclear factor kappa B (NF-*κ*B).

NF-*κ*B is a transcription factor, implicated in the expression of over 100 proinflammatory genes which mostly participate in the host immune response [2, 8]. NF-*κ*B is exploited by many viruses such as RV and retroviruses to promote their replication by preventing viral-induced apoptosis and evading the immune system [8, 9]. 

Previous studies have found that RV can activate NF-*κ*B via I*κ*B kinase (IKK)-*α*/*β* or phosphorylation of I*κ*B and cause the upregulation of numerous cytokines [2, 10, 11]. 

Despite the vast array of asthma medication available, *β*_2_ agonists and corticosteroids remain the most effective treatments in asthma to control and prevent symptoms [12]. However their use does not prevent asthmatics from respiratory viral infections or RV-induced asthma exacerbation [13]. Therefore there is a need for specific treatment strategies for RV-induced asthma exacerbation. 

Studies have shown that inhibition of NF-*κ*B reduces viral-induced cytokine release and also inhibits replication of human immunodeficiency virus (HIV) and human cytomegalovirus (HCMV) [14, 15]. However to date no research has examined the effect of inhibiting NF-*κ*B on RV-induced cytokine release and RV replication in airway cells. For this reason, this study investigated if NF-*κ*B inhibitors, either alone or in combination with the currently used asthmatic drugs, *β*_2_ agonists and corticosteroids, reduce both RV-induced cytokine release and RV replication in human primary airway fibroblasts. This study may provide *in vitro* data that may be beneficial in determining a more adequate treatment regimen for RV-induced asthma exacerbations.

## 2. Materials and Methods

### 2.1. Isolation and Culture of Human Fibroblasts

Primary human airway fibroblasts were obtained from macroscopically healthy lung tissue. Lung tissue was obtained from patients undergoing resections or transplantations (see Table 1 for demographics). 

Parenchymal tissue was washed in sterile Hanks balanced salt solution (Trace Scientific, Melbourne, Australia), minced and suspended in Dulbecco's Modified Eagle's Medium (DMEM) (Sigma-Aldrich, Castle Hill, Australia) supplemented with 10% (v/v) foetal bovine serum (FBS) (JRH Biosciences, Melbourne, Australia), 20 U/mL penicillin, 20 g/mL streptomycin, and 2.5 g/mL amphotericin B (Invitrogen, Mount Waverley, Australia) in 75 cm^2^ tissue culture flasks. The cells were grown to confluence, and fibroblast characteristics were confirmed by normal fibroblast growth patterns and cell morphology as described previously by Ghildyal et al. [7]. All experiments were carried out with fibroblasts between passages 2 and 8. 

Ethical approval for all experiments involving the use of human lung tissue was provided by The University of Sydney Human Ethics Committee and the Sydney South West Area Health Service, and written informed consent was obtained.

### 2.2. RV Propagation and Ultraviolet Inactivation of RV (UVi-RV)

Major group human RV serotype-16 was purchased from ATCC (Manassas, USA) and propagated in Ohio HeLa cells as previously described by Papi and Johnston [16]. In some experiments RV was UV inactivated in 24 well plates containing 200 *μ*L of RV/well at a distance of 5 cm from a 30 W UV light source (germicidal lamp G30T8, Sankyo Denki, Japan) for 15 minutes. UV inactivation was established to be effective by a RV titration assay and was used as a noninfectious virus control.

### 2.3. Drug Concentrations

Dexamethasone, BAY-117085 (BAY), dimethyl fumarate (DMF) (Sigma-Aldrich), fluticasone propionate, and salmeterol (GSK, Boronia, Australia) were dissolved in dimethyl sulfoxide (DMSO) (Sigma-Aldrich) at 10^−3^ M and further diluted in 0.1% FBS/antibiotics/DMEM to give final experimental concentration ranges of 10^−12^–10^−6^ M and corresponding vehicle controls 0.001–0.1% DMSO. For drug combination experiments, the lowest concentration of drug to produce an effect was used.

### 2.4. RV Infection of Primary Human Airway Fibroblasts

Fibroblasts were seeded at 3.2 × 10^4^ cells/mL into 6 well plates in 10% FBS/DMEM and grown for 3 days. Prior to RV infection, a cell count was carried out to determine the amount of RV (or UVi-RV) needed to infect at a multiplicity of infection (MOI) of 0.1. The medium was then replaced with 0.1% FBS/antibiotics/DMEM and or drug/vehicle and incubated for 1 hour at 37°C and 5% CO_2_. Some of the wells were infected at an MOI of 0.1 with UVi-RV or live RV respectively and left for 1 hour at 37°C and 5% CO_2_. Plates were rocked every 15 minutes to disperse the virus or contents. The medium was removed, the cells were washed with the Hanks solution, and 2 mL/well of fresh sterile 0.1% FBS/antibiotics/DMEM or drug/vehicle was added. The plates were then incubated at 37°C and 5% CO_2_ over time, and supernatants were collected and stored at −20°C prior to analysis using ELISA and RV titration assays.

### 2.5. RV Titration Assay

All viral concentrations were measured by a titration assay as outlined by Papi and Johnston [16]. Briefly, RV levels were determined by serially titrating log-diluted concentrations of the cell-free supernatant in quadruplicates on the Ohio HeLa cells. The Ohio HeLa cells were seeded at a concentration of 2 × 10^4^ cells/mL in 96 well plates (150 *μ*L/well) and then 50 *μ*L of supernatants with RV or control medium was added to the wells. The plates were rocked at 100 rpm for 15 minutes at room temperature before being cultured for 5 days at 37°C and 5% CO_2_. After 5 days of culture, the cytopathic effect (CPE) was assessed by comparing the cells in the RV-infected wells to the control wells. Viral concentration was determined using Karber's method as described previously [7]. The concentration of RV-16 stock was determined to be 6.3 × 10^5^ virions/mL.

### 2.6. ELISA

ELISA kits for IL-28A (interferon *λ*_2_), IL-29 (interferon *λ*_1_), IL-6, and IL-8 were purchased from R&D Systems (Minneapolis, USA) and BD Biosciences (North Ryde, Australia), respectively. ELISAs were carried out according to the manufacturer's instructions. The detection limits of these assays were 62.5 pg/mL (IL-28A), 31.2 pg/mL (IL-29), 7.8 pg/mL (IL-6), and 15.6 pg/mL (IL-8).

### 2.7. Statistical Analysis

Since there were no differential responses to RV in cells from patients with different diagnoses, results were pooled and analysed together in this study. All data were verified for normality and values presented as mean ± SEM. When results were nonparametrically distributed, the dataset was log transformed prior to statistical analysis using GraphPad Prism Version 5 software (Calif, USA). ELISA and RV titration results were analysed by either a 1-or 2-way analysis of variance (ANOVA) with the Dunnett or Bonferroni posttest comparisons where appropriate and indicated. Statistical significance was shown when *P* ≤ 0.05.

## 3. Results

### 3.1. RV Infects Human Primary Airway Fibroblasts and Stimulates IL-6 and IL-8 Production but Not IL-28A (Interferon *λ*_2_) and IL-29 (Interferon *λ*_1_)

To determine if RV induced IL-6 and IL-8 release and replicated in fibroblasts, tissue culture medium of infected fibroblasts was assessed using RV titration assay and ELISA at 0, 3, 6, 24, 48, and 72 hours post infection. 

**Figure 1:**
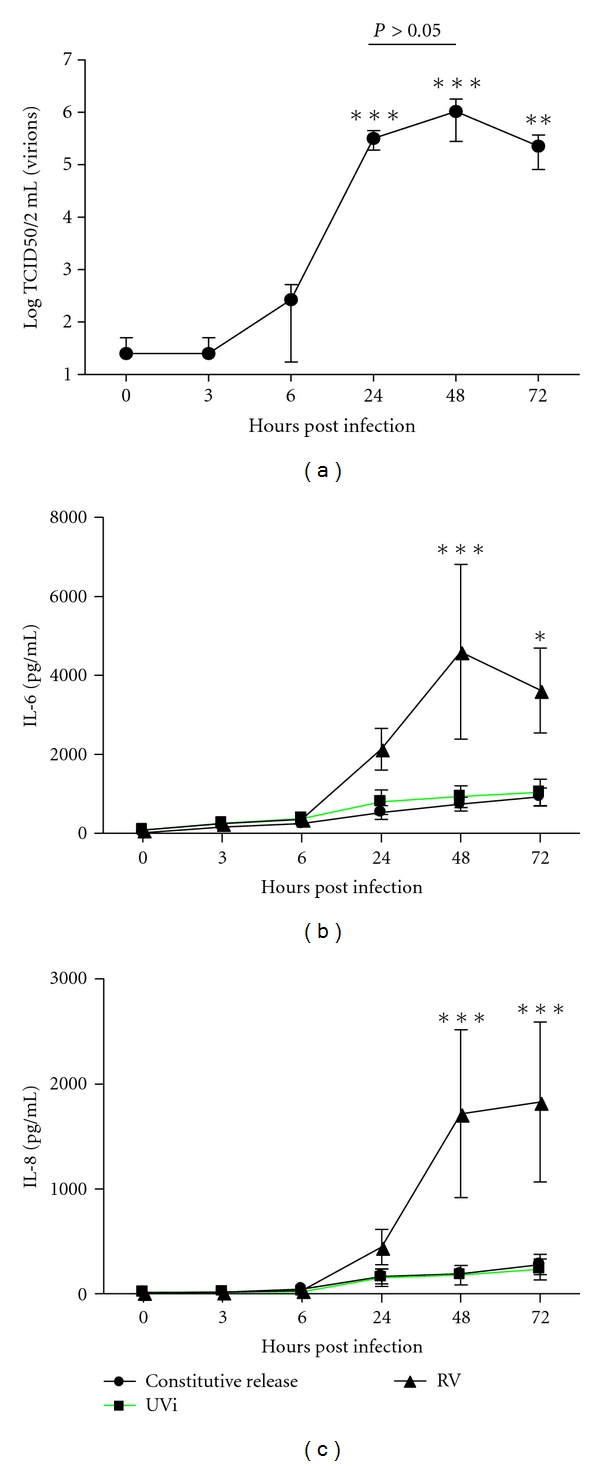
(a) Time course of RV replication. Concentration is of RV from infected fibroblasts (MOI = 0.1) at 0, 3, 6, 24, 48 and 72 hours post infection were measured by RV titration. RV concentration was compared with each time point post infection using a 1-way ANOVA (*n* = 5). (b,c) Time course of RV-induced IL-6 and IL-8. Concentration of (b) IL-6 and (c) IL-8 release from noninfected fibroblast (constitutive release) or UVi-RV-(UVi-) or RV-16-(RV-) infected fibroblasts (MOI = 0.1) at 0, 3, 6, 24, 48 and 72 hours post infection were measured by ELISA. RV-induced IL-6 and IL-8 at 48, and 72 hours post infection compared to control and UVi (2-way ANOVA, *n* = 5). All data are presented as mean ± SEM. Significance of comparisons is represented as *P < 0.05, **P < 0.01, and ***P < 0.0001.

RV replicated in fibroblasts and was maximal 24h post infection when compared to 0 hours (*P* < 0.0001; *n* = 5; Figure 1(a)). There was no statistical significance between the number of virions at 24 and 48 hours post infection.

As can be seen in Figures 1(b) and 1(c), RV-induced IL-6 and IL-8 were maximal at 48 hours, compared to respective constitutive release (*n* = 5, *P* < 0.0001). No induction was observed with UVi-RV. 

RV-16 did not induce IL-28A and IL-29 from human primary airway fibroblasts (*n* = 5, data not shown).

### 3.2. Corticosteroids Suppress and *β*_2_ Agonists Increase Primary Airway Fibroblast Responses to RV Infection

To determine if the corticosteroids dexamethasone and fluticasone and the *β*_2_ agonist salmeterol could inhibit RV-induced IL-6 and IL-8 and RV replication, tissue culture medium from fibroblasts pretreated with drug for 1 hour and then infected with RV was analysed by ELISA after 48 hours and RV titration 24 hours post infection.

**Figure 2:**
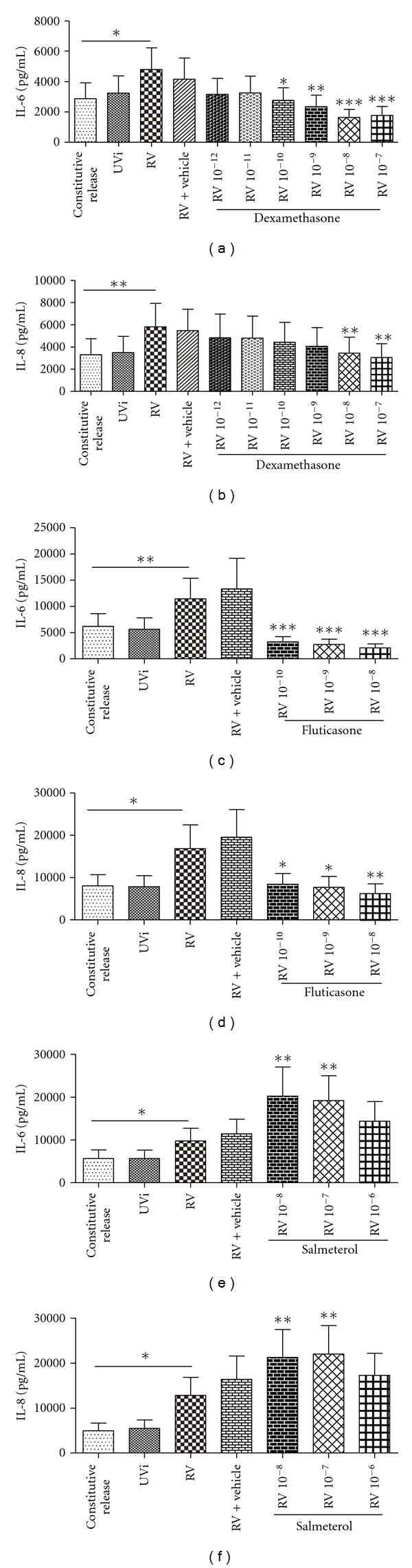
(a–f) Effect of dexamethasone (Dex), fluticasone (Flut) and salmeterol (Sal) on RV-induced IL-6 and IL-8. Concentration of IL-6 and IL-8 release from noninfected fibroblasts (constitutive release), UVi-RV-(UVi-) or RV-16-infected fibroblasts (RV) (MOI = 0.1), highest concentration of vehicle (Dex & Sal: 0.1% DMSO; Flut: 0.001% DMSO) and RV infected fibroblasts in the presence of Dex: 10^−12^–10^−7^ M (*n* = 7), Flut: 10^−10^–10^−8^ M (*n* = 7) and Sal: 10^−8^–10^−6^ M (*n* = 9) were measured 48 hrs post infection by ELISA. All IL-6 and IL-8 concentrations were compared to their respective RV-induced values (in the absence of drug and vehicle), using a 1-way ANOVA. All data are presented as mean ± SEM. Significance is represented as *P < 0.05, **P < 0.01, and ***P < 0.0001.

As before RV induced IL-6 and IL-8 (Figure 2, *n* = 7–9, *P* < 0.05). Dexamethasone significantly inhibited both RV-induced IL-6 and IL-8 at concentrations greater than 10^−10^ M and 10^−8^ M, respectively (Figures 2(a) and 2(b), *n* = 7, *P* < 0.05). Fluticasone significantly inhibited both RV-induced cytokines at all concentrations tested 10^−10^–10^−8^ M (Figures 2(c) and 2(d), *n* = 7, *P* < 0.05). Dexamethasone did not inhibit the constitutive release of IL-6 and IL-8 at the concentrations tested (*n* = 7, *P* > 0.05), while fluticasone inhibited the constitutive release of IL-6 and IL-8 at all concentrations (10^−10^–10^−8^ M; *n* = 7, *P* < 0.05) (Table 2). However salmeterol further increased RV-induced IL-6 and IL-8, almost 2-fold more than RV control at concentrations 10^−8^ to 10^−7^ M (Figures 2(e) and 2(f), *n* = 9, *P* < 0.05). Salmeterol significantly induced the constitutive release of IL-6 at 10^−8^ M and IL-8 at 10^−8^ and 10^−7^ M, (Table 2, *n* = 9, *P* < 0.05). The highest concentration of vehicle used had no significant effect on the level of IL-6 and IL-8 induction. Dexamethasone, fluticasone, and salmeterol did not alter RV replication (data not shown).

### 3.3. NF-*κ*B Inhibitors Increase RV Replication and Suppress RV-Induced IL-6 in Primary Airway Fibroblasts

To determine if the NF-*κ*B inhibitors, BAY and DMF could inhibit RV-induced IL-6 and IL-8 and RV replication, tissue culture medium from fibroblasts pretreated with drugs and then infected with RV was analysed by ELISA after 48 hours and RV titration 24 hours post infection.

**Figure 3:**
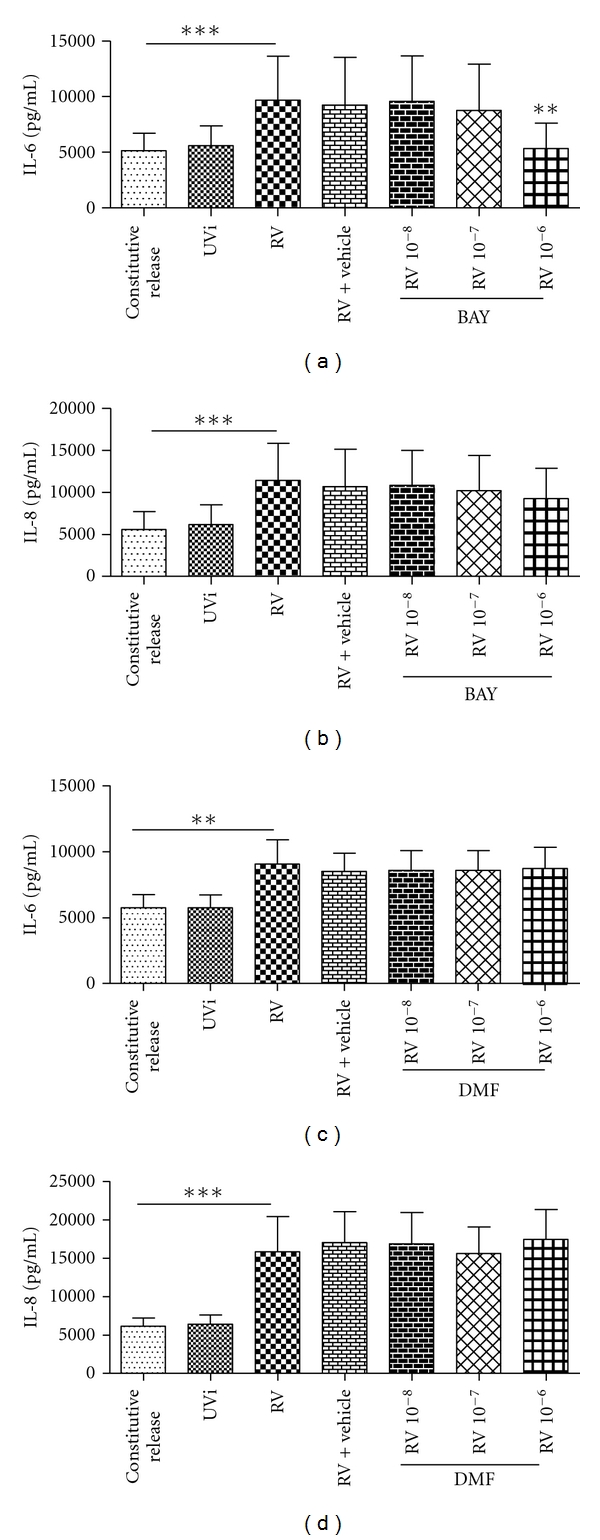
(a–d) Effect of BAY and DMF on RV-induced IL-6 and IL-8. Concentration of IL-6 and IL-8 release from noninfected fibroblast (constitutive release), UVi-RV-(UVi-) or RV-16-infected fibroblasts (RV) (MOI = 0.1), highest concentration of vehicle (0.1% DMSO) and RV infected fibroblasts in the presence of 10^−8^–10^−6^ M BAY (*n* = 10) and DMF (*n* = 9) measured 48 hrs post infection by ELISA. All IL-6 and IL-8 concentrations were compared to their respective RV-induced values (in the absence of drug and vehicle), using a 1-way ANOVA. All data are presented as mean ± SEM. Significance is represented as **P < 0.01 and ***P < 0.0001.

As before RV induced IL-6 and IL-8 (Figure 3, *P* < 0.05, *n* = 9-10). BAY significantly inhibited the constitutive release (Table 3) and RV-induced IL-6 at 10^−6^ M but failed to inhibit IL-8 at the concentrations used (Figures 3(a) and 3(b), *n* = 10, *P* < 0.05). DMF had no effect on RV-induced IL-6 and IL-8 (Figures 3(c) and 3(d), DMF: *n* = 9). Interestingly, DMF increased the constitutive release of IL-8 (Table 3, *n* = 9, *P* < 0.05). The highest concentration of vehicle used to dissolve BAY and DMF had no effect on the level of IL-6 and IL-8 induction.

**Figure 4:**
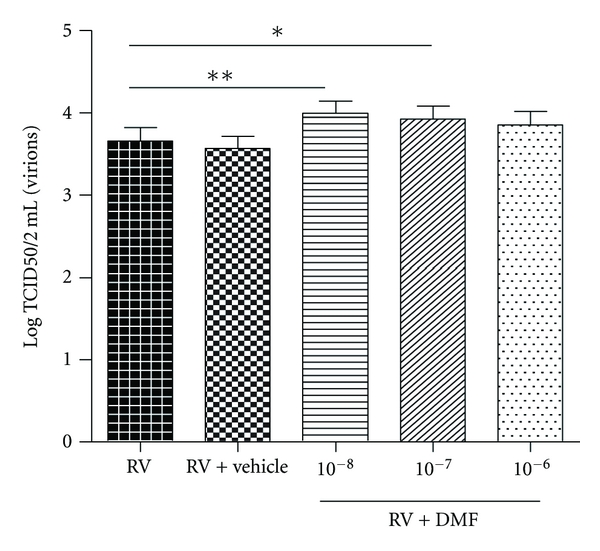
Effect of DMF on RV replication. Concentration of virus from RV-infected fibroblasts ± vehicle (0.1% DMSO); or 10^−8^–10^−6^ M DMF (*n* = 14) was measured 24 hrs post infection by RV titration. All RV concentrations were compared to RV concentration in the absence of drug and vehicle by 1-way ANOVA. All data are presented as mean ±SEM. Significance is represented as *P < 0.05 and **P < 0.01.

BAY had no effect on RV replication (*n* = 10, data not shown). DMF, however, significantly increased RV replication at 10^−8^–10^−7^ M (Figure 4, *n* = 14, *P* < 0.05). The highest concentration of vehicle used to dissolve BAY and DMF had no effect on RV replication.

### 3.4. Addition of a NF-*κ*B Inhibitor to the Combination Therapy of a Corticosteroid and *β*_2_ Agonist Inhibits RV-Induced IL-6 in Primary Airway Fibroblasts

Since BAY was able to inhibit RV-induced IL-6 release but not IL-8, BAY at 10^−6^M was combined with the lowest concentration of salmeterol (Sal) (10^−8^ M) and fluticasone (Flut) (10^−10^ M) which caused an effect, to examine if the combination could inhibit RV-induced IL-6 and RV replication.

**Figure 5:**
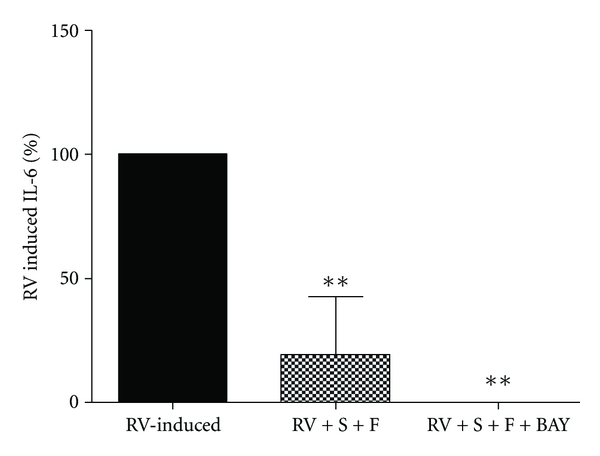
Salmeterol + fluticasone (Sal + Flut) and Sal + Flut + BAY inhibit RV-induced IL-6: the amount of IL-6 induced from fibroblasts infected with RV-16 (MOI = 0.1) was expressed as 100%. Inhibition caused by drug combinations, Sal + Flut (10^−8^ + 10^−10^ M) and Sal + Flut + BAY (10^−8^ + 10^−10^ + 10^−6^ M) (for all *n* = 4) was measured 48 hrs post infection by ELISA and expressed as a percentage of RV-induced IL-6. Percentage inhibition caused by drugs was compared using 1-way ANOVA with RV-induced IL-6. All data are presented as mean ± SEM. Significance is represented as **P < 0.01.

As before RV induced IL-6 (Figure 5, *n* = 4, *P* < 0.05). Salmeterol and fluticasone in combination (Sal  +  Flut) inhibited RV-induced IL-6 by 80%, and in the additional presence of the NF-*κ*B inhibitor BAY (Sal  +  Flut  +  BAY), total inhibition occurred (100%) (Figure 5, *n* = 4, *P* < 0.01). However RV replication was not altered (*n* = 4, data not shown). The highest concentration of vehicle used to dissolve Sal  +  Flut and Sal  +  Flut  +  BAY had no effect on IL-8 induction or RV replication (data not shown).

## 4. Discussion

This study examined the effects of current asthma medications such as corticosteroids and *β*_2_ agonists and potential novel treatments such as NF-*κ*B inhibitors, as well as their combination in the treatment of RV-induced inflammation and RV replication in airway fibroblasts. The study confirmed that RV was able to infect and replicate in primary airway fibroblasts and that RV can induce proinflammatory cytokines IL-6 and IL-8 but not IL-28A and IL-29 from primary airway fibroblasts. Therefore this *in vitro* model simulates a possible underlying inflammatory scenario experienced during RV-induced asthma exacerbations *in vivo.* No induction was observed with UVi-RV, and thus UVi-RV-treated fibroblasts were not studied further.

Interferons are cytokines which are released by cells in response to pathogens to trigger protective defences of the immune system, and it has been shown that asthmatic patients may be more susceptible to RV infection due to their deficient interferon *β* and *λ* responses in bronchial epithelial cells [5, 17]. In our study we measured both IL-28A and IL-29 (2 members of the interferon-*λ* family) and found that RV infection of primary human fibroblasts does not induce interferon-*λ* above the level of constitutive release. This indicates that the production of interferons in response to RV is cell type specific, and our results are similar to other findings that showed RV does not induce interferon *β* in primary human fibroblasts [18], supporting their hypothesis that this susceptibility to RV infection may cause fibroblasts to act as reservoirs for RV replication and spread to the lower airways.

Toll-like (TLR) receptors are a class of receptors which recognise distinct molecular patterns that are shared by pathogens but not by the host. RV is a single-stranded RNA virus, which in theory could be detected by both TLR 7/8 (single-stranded RNA) and TLR 3 (double-stranded RNA) as replication occurs. In fibroblasts the mechanism by which RV induces cytokines is not known. In our experiments UVi-RV did not induce cytokines, suggesting that cytokine induction is replication dependent (i.e., the cell is detecting and responding to double-stranded RNA). Similarly, in bronchial epithelial cells, RV induces cytokines via the activation of TLR-3 and not TLR 7/8 [19]. However, this response is likely specific to RV as in our previous studies we have shown that fibroblasts respond to agonists of TLR-3 and TLR 7 and 8 [20]. TLR signalling pathways have not been extensively studied in lung fibroblasts; however, their activation leads to downstream activation of transcription factors such as NF-*κ*B, and this results in the upregulation and induction of various inflammatory cytokines such as IL-6 and IL-8 [21].

The study showed that, at a concentration of 10^−10^ M, fluticasone inhibited both RV-induced IL-6 and IL-8, while dexamethasone inhibited only IL-6. Fluticasone is a more potent corticosteroid in inhibiting inflammation [22], and our data reflect this fact. It is also interesting to note that, although IL-8 is released more abundantly than IL-6 [23], this selective inhibition suggests that IL-8 may at least in part be steroid insensitive, and other studies have also demonstrated that certain cytokines are steroid insensitive [24, 25]. Nevertheless, to our knowledge this study is the first to report of RV-induced IL-6 and IL-8 inhibition by corticosteroids in primary airway fibroblasts and confirms previous studies demonstrating that corticosteroids inhibits RV-induced cytokines [7, 26]. 

The major function of both long- and short-acting *β*_2_ agonists in the treatment of asthma is to maintain or to induce airway relaxation. *β*_2_ adrenoceptors are present on lung fibroblasts as well as airway smooth muscle (ASM); therefore, *β*_2_ agonists may affect fibroblast activities [27]. Previous studies have produced conflicting results in regards to the effects of *β*_2_ agonists on cytokine induction in various airway cells. *β*_2_ agonists increased the secretion of TNF-*α*- and TGF-*β*-induced IL-6 in ASM, had no effect on RV-induced IL-8 in bronchial epithelial cells, and inhibited IL-4 in human peripheral blood mononuclear cells and inhibited cytokine-induced adhesion molecule expressions, such as ICAM-1 in human lung fibroblasts [25–31]. This suggests that *β*_2_ agonists *in vitro* can have positive, neutral, or even negative effect on cytokine induction in various airway cells and that the effects of *β*_2_ agonists may be stimulus and cell type dependent. The current study showed for the first time that *β*_2_ agonists further increased RV-induced IL-6 and IL-8. The mechanism by which *β*_2_ agonists increase inflammation may be explained by their mechanism of action at the cellular level. *β*_2_ agonists stimulate the *β*_2_ adrenoceptor and activate adenyl cyclase which gives rise to an increase in intracellular cAMP levels which binds to the cAMP responsive element binding protein (CREB) in the promoter region of genes and can result in upregulation of various genes [29]. Since RV infection alone induces cytokines, the use of *β*_2_ agonists may result in a second signal to further induce proinflammatory cytokines. 

Despite their inflammatory modulatory capacity, neither corticosteroids nor *β*_2_ agonists affected RV replication and this suggests that RV replication may not be dependent on RV-induced inflammation. However *in vivo* studies have shown that intranasal use of corticosteroids increased and prolonged RV number and shedding which may be due to the presence of the immune system [32]. 

There is good evidence suggesting that RV-induced inflammation is due to NF-*κ*B activation [6, 33]. For this reason, this study is the first to have examined the effects of inhibiting NF-*κ*B on RV-induced IL-6 and IL-8 and RV replication.

BAY inhibits NF-*κ*B by inhibiting I*κ*B-*α* phosphorylation [34]. The study showed that BAY inhibited RV-induced IL-6 but not IL-8 and this was unexpected as previous studies found that transcription of IL-6 and IL-8 is regulated by the same transcription factors: NF-*κ*B, AP-1, CREB protein, and CCAAT/enhancer binding protein (C/EBP) [25, 35]. Although transcription of IL-8 is mediated by the same transcription factors as IL-6, although IL-8 is partially regulated by NF-*κ*B, it may be more dominantly or synergistically regulated by other transcription factors such as AP-1 or C/EBP and hence explains the result [16, 36]. This study showed that the inhibitory effects of BAY on RV-induced IL-6 and IL-8 were not as effective as the corticosteroids.

DMF inhibits the translocation and partially inhibits the transactivation of NF-*κ*B but does not inhibit NF-*κ*B completely [37]. In our study, DMF had no effect on RV-induced IL-6 and IL-8 but increased RV replication. The increase in replication may be an example of how some viruses can exploit NF-*κ*B for their own replication and survival [8]. Most viruses that induce NF-*κ*B activity often harbour NF-*κ*B binding elements in their viral promoters and therefore would have a replicative advantage if there is active NF-*κ*B in the cytoplasm [8, 38]. One example of this is the low-level NF-*κ*B activation by HIV-1 which allows HIV-1 to maintain a chronic infection in myeloid cells [38]. By using the basic local alignment search tool (BLAST) we found that there are a few NF-*κ*B binding motifs on the RV genome [39]. Although we cannot confirm whether these binding motifs can actively bind NF-*κ*B and produce a functional outcome, if they are true it may be possible that RV-16 may be utilising a similar process to HIV-1. Alternatively, DMF may have other properties which may be delaying cellular apoptosis and therefore allowing increased replication to occur [40], both, which require further investigation. 

These results suggest that NF-*κ*B activation alone may not be the factor that results in RV-induced inflammation but perhaps a combined activation of NF-*κ*B, AP-1 and C/EBP, and alternative inhibitors of these transcription factors should be examined.

This study also examined whether the commonly used corticosteroid and *β*_2_ agonist combination therapy in the treatment of asthma could affect RV-induced IL-6 and RV replication. Our study showed that fluticasone inhibited IL-6 production (100%) in response to RV infection, whilst salmeterol increased RV-induced IL-6 production. However when used together suppression of RV-induced IL-6 occurred (80%), reflecting similar results to Edwards et al., [2] showing the same combination inhibiting RV-induced IL-8 from bronchial epithelial cells. The addition of the NF-*κ*B inhibitor BAY to this combination suppressed RV-induced IL-6 further (100%). Despite neither combination altering RV replication, the results were as expected, as earlier we established that both BAY and fluticasone were potent inhibitors of RV-induced IL-6, and this combined result shows the additive inhibitory effect of these drugs. These findings suggest that during RV-induced asthma exacerbations, the use of combination therapy may not be as useful as corticosteroids alone, but may still be beneficial. Furthermore, it is possible that incorporating a NF-*κ*B inhibitor into this combination may make the combined therapy more effective for the treatment of both asthma and RV-induced asthma exacerbations.

This study albeit *in vitro* suggests that in the event of RV-induced asthma exacerbation, asthma steroidal medication alone is more beneficial in inhibiting RV-induced inflammation than *β*_2_ agonists. In reality this is unrealistic as *β*_2_ agonists are required to provide bronchorelaxation during RV-induced asthma exacerbations. Therefore perhaps alternative bronchodilators such as anticholinergics could be used during a viral exacerbation. Furthermore, NF-*κ*B inhibitors are not as effective as corticosteroids and may be detrimental if used incorrectly. In conclusion, NF-*κ*B inhibitors remain a potential therapeutic treatment for RV-induced asthma exacerbation; however, future research into drug combinations such as corticosteroids and NF-*κ*B inhibitors, multiple NF-*κ*B inhibitors or inhibitors of other transcription factors such as AP-1 and C/EBP may pave the way for more therapeutic options.

## Figures and Tables

**Table 1:** Demographics of donors from whom fibroblasts used in this study were isolated.

Patient	Disease	Sex	Age (years)
1	Transposition of the great arteries	M	39
2	Chronic obstructive pulmonary disease (COPD)	M	56
3	No disease	M	48
4	Idiopathic pulmonary fibrosis (IPF)	M	61
5	Emphysema	M	63
6	Small cell carcinoma	M	63
7	Lymphangioleiomyomatosis (LAM)	F	51
8	Pulmonary fibrosis	M	53
9	Bronchiectasis	M	53
10	Emphysema	M	40
11	Non-small cell carcinoma	M	77
12	Cystic Fibrosis	M	45
13	Non Small Cell Carcinoma	F	63
14	Non-small cell carcinoma	F	79
15	Primary pulmonary hypertension	F	36
16	Lymphangioleiomyomatosis (LAM)	F	33
17	Emphysema	F	56
18	Emphysema	F	48
19	Melanoma	M	63
20	Carcinoma	M	59
21	Lesion	F	58
22	Resection	M	48
23	Carcinoma	F	83
24	Idiopathic pulmonary fibrosis (IPF)	M	57
25	Carcinoma	F	76
26	Emphysema	F	50
27	Idiopathic pulmonary fibrosis (IPF)	M	56
28	Pulmonary fibrosis	M	68
29	Rejection:pneumonitis	M	21
30	*α*1 antitrypsin deficiency	M	55
31	Hypersensitive pneumonitis	M	59
32	*α*1 antitrypsin deficiency	M	42
33	Emphysema	M	42
34	Bronchiectasis	M	39
35	Small cell Carcinoma	F	78

**Table 2:** Effects of dexamethasone (Dex), fluticasone (Flut), and salmeterol (Sal) on the constitutive release of IL-6 and IL-8.

Constitutive release [pg/mL]				10*^x^* [M]						
Drug	Cytokine	Constitu-tive release	Vehicle	−12	−11	−10	−9	−8	−7	−6
Dex	IL-6	2874 ± 1033	2974 ± 1078	3295 ± 1092	3734 ± 1369	3387 ± 913.8	2729 ± 834.1	1576 ± 567.4	1264 ± 380.6	
	IL-8	3305 ± 1429	3424 ± 1534	3277 ± 1521	3376 ± 1548	3499 ± 1391	3206 ± 1220	2590 ± 1240	2492 ± 1182	

Flut	IL-6	6195 ± 2429	6187 ± 2400			1712 ± 545.8***	1286 ± 375.0***	1123 ± 355.5***		
	IL-8	8035 ± 2609	8639 ± 2977			3334 ± 988.1*	2565 ± 695.6**	2681 ± 763.0*		

Sal	IL-6	5646 ± 2009	6620 ± 2438					9440 ± 4112*	7903 ± 2955	6277 ± 2335
	IL-8	4962 ± 1698	5974 ± 2023					8186 ± 3583**	6122 ± 2226*	5196 ± 1807

Values are means ± SEM.

*P < 0.05, **P < 0.01, and ***P < 0.0001.

**Table 3:** Effects of BAY and DMF on the constitutive release of IL-6 and IL-8.

Constitutive release [pg/mL]				10*^x^* [M]		
Drug	Cytokine	Noninfected	Vehicle	−8	−7	−6
BAY	IL-6	5159 ± 1531	5005 ± 1631	5826 ± 1879	4790 ± 1530	2556 ± 803.9**
	IL-8	5598 ± 2109	4573 ± 1947	4442 ± 1761	3958 ± 1719	3423 ± 1334

DMF	IL-6	5743 ± 1001	5970 ± 1226	5907 ± 1086	5998 ± 1122	5937 ± 984.7
	IL-8	6151 ± 1055	7296 ± 1803	7180 ± 1664	7151 ± 1756	8329 ± 1780*

Values are means ± SEM.

*P < 0.05, and ***P < 0.0001.
